# Christian Science Outdone

**Published:** 1889-05

**Authors:** 


### CHRISTIAN SCIENCE OUTDONE.

C. D. Towt is or was a stock broker in Broad Street, New York City, where he speculated for the matter of a dozen years.

C. D. Towt is an enthusiast in the matter of the Christian religion as he interprets it, and for a long time his steel-plate checks upon his banker, were adorned with a head of Christ, with the underlined words, "The Lord is my Redeemer."

Whether it was that his conscience smote him for advertising Christ as the redeemer of his checks, in view of the dubious nature of his business, or whatever the cause, C. D. Towt subsequently changed the underlining to "The Lord is my Shepherd, I shall not want." So far as we know, he, the said, C. D. Towt, did not want sheep to be sheared, but somehow the fleeces didn't pan out as they were expected to, for only the other day this "Christian at work," suspended payment. Perhaps some sinful wolves, not holding christianity and money changing of the same piece, broke into C. D. Towt's sheep fold, and knocked things silly. It will be curious to observe under what assuring motto, C. D. Towt, will resume business. We would suggest the following in imitation of Tittlebat Titmouse, of "Ten Thousand a Year" notoriety :

Towt, C. D., Sir, is my name,
Broad street is my station,
Speculation is my fame,
And Christ is my salvation.

